# Essay on Pyelitis Calculosa, or Stone in the Kidney

**Published:** 1848-10

**Authors:** James Bryan

**Affiliations:** Philadelphia, Member of the American Medical Association, Professor of Surgery in Geneva Medical College, N. Y., &c., &c.


*An Essay on Pyelitis Calculosa, or Stone in the Kidney. Translated from the French of P. Rayer, Physician to the Hospital de la Charité, &c., &c., &c.—with notes and additional cases.* By JAMES BRYAN, M. D., of Philadelphia, Member of the American Medical Association, Professor of Surgery in Geneva Medical College, N. Y., &c., &c.

Pyelitis, (from πυελος pelvis,) or inflammation of the pelvis and calices of the kidney, is a disease distinct from nephritis proper, not only in its seat and anatomical characters, but also in its symptoms and accompanying conditions.

Pyelitis is sometimes acute, but more frequently chronic; it may be divided into several distinct species, according to the character of the inflammation, or the nature of the causes which produce it. (Pyelitis simplex, P. calculosa; P. blennorrhagica, and P. gangrenosa.)

It may attack the whole or a part of one pelvis, or of both, or only one or more calices.

*General Characters.*—The following alterations occur in acute pyelitis.

The mucous membrane of the pelvis and of the calices is more or less injected—this membrane presents a general redness, from a vascular network, formed by a large number of vessels, invisible in a healthy condition.

At other times red spots are seen on the membrane, formed by a mesh of small vessels almost invisible to the naked eye.

These vascular spots are sometimes very small, and then appear like red points disseminated over the mucous membrane. Besides these, small petechiæ are sometimes seen like true ecchymosis; at other times a quantity of coagulated blood, alone or mixed with urine, is found in the pelvis and calices. These petechiæ, ecchymosis or deposits of blood, are observed, particularly in gangrenous pyelo-nephritis, produced by morbid poisons, as variola or malignant scarlatina, either petechial or hæmorrhagic.

In acute inflammation of the pelvis we occasionally find the internal surface of the mucous membrane deeply injected, covered here and there with partial deposits of pseudo-membrane, more or less adherent, of a whitish or greyish color.

In the acute form of inflammation of the pelvis, the mucous membrane is not much thickened; there is not much contraction either of the orifices of the pelvis, or of its interior, or of the mouth of the ureter; while in pseudo-membranous acute pyelitis, these orifices may be contracted or entirely closed.

When an acute pyelitis follows a retention of urine, the pelvis and calices are always more or less dilated, and the cones, to some extent, absorbed. This dilatation of the pelvis and of the calices, with diminution of the cones, is particularly obvious in chronic pyelitis.

Softening, ulceration and perforation of the pelvis have been observed only in rare cases of calculous pyelitis, become acute, or in pyelitis originally gangrenous.

The submucous tissue of the pelvis and of the calices is sometimes infiltrated with serosity or pus, in severe acute pyelitis : the renal tissues then present inflammatory lesions.

In acute pyelitis, the urine in the pelvis and the calices, is always mixed with a quantity of purulent mucus or pus, or even blood. When the mucus, pus or blood is not visible with the naked eye, we may detect, by means of the microscope, globules of pus and those of blood. In reference to the chemical and physical properties of the urine, they are not all alike in the various forms of pyelitis ; sometimes the urine contains the urates, in amorphous powder, sometimes uric acid crystals, sometimes crystals of an amoniaco-magnesian phosphate, or finally, albumen.

The most common appearance of the mucous membrane of the pelvis in chronic pyelitis, old and well marked, is the tint, either general or partial, of a dead white. The redness seen in some chronic cases is never so decided as that seen in acute cases ; the vascular interlacement is not so distinct, but the principal vessels of which it is composed appear frequently large, and as though varicose.

The veins which course over the exterior of the kidney, sometimes attain a great size, and form large rolls around the pelvis and ureter.

The mucous membrane of the pelvis and of the calices presents often, to a greater or lesser extent, a brownish red tint, and the vessels which pass along the surface appear of a decidedly sooty color.

In very old chronic pyelitis, it is not uncommon to observe in the pelvis, a number of grey or redish patches.

In chronic pyelitis, with enlargement of the pelvis and calices, by pus or purulent urine, the pelvis and the species of pouches formed by the dilated calices, are, on the internal surface, of a dead white, very different from the bluish white semitransparent tint, natural to the parts. The mucous membrane, somewhat thickened, does not exhibit vessels on either surface.

The thickening of the mucous membrane is sometimes so great that the orifices from the calices to the pelvis are extremely contracted ; these openings are sometimes changed into fibrous cords.

I have sometimes observed, in chronic pyelitis, and less frequently in acute pyelitis, on the inflamed surface of the mucous membrane, a transparent vesicular eruption, the size of a pin's head, containing an aqueous fluid ; this is seen also under other circumstances.

The result of ulceration of the pelvis and calices may be, the destruction of the surface or substance of these parietes, in one or more places, as when gangrene attacks them. Fistulæ renales are then established, which communicate with the sub-peritoneal cellular tissue with the cavity of the peritoneum, with the large intestine or duodenum, &c. The infiltrated urine produces either perineal inflammation, peritonitis, vomiting of urine, or is discharged per anum mixed with pus.

These accidents may occur without any true dilatation of the calices or pelvis, or preternatural enlargement of the kidneys. Nevertheless, it is only generally after atrophy of the substance of the kidney, and the alteration of the pelvis and calices into a multilocular pouch, filled with purulent pus, that fistulæ renales are established.

Ulceration of the pelvis and calices may cicatrize. Some cicatrices appear as simple irregular depressions of the surface, of a greyish colour, which may be covered by a pin's head ; others of greater size are of a dead white or pearl (nacré) colour, and exhibit depressions and salient lines converging or stellated.

In chronic pyelitis, with obstruction to the passage of the urine, the pelvis and calices become dilated, and the substance of the kidney becomes atrophied. Becoming more and more distended, the pelvis and calices are finally transformed into a multilocular pouch, whose subdivisions communicate with each other through the pelvis, sometimes very much dilated. The relations of this pouch with the neighbouring organs, vary according to its dimensions. The right kidney dilated, may push the liver towards the chest, and become adherent to it ; the pus or purulent urine in the renal pouch may be directed to the liver, through one or more openings under this organ, and in this way be forced into an abscess of the liver, or even one of the bronchi. Communications may be made with the duodenum. Below, it may pass behind the coecum or extend to the crural arch. In some cases the dimensions of these pouches may be accurately made out by the aid of the touch, and by percussion.

When inflammation attacks the pelvis and calices of the left kidney, it may be dilated upwards, contract adhesions with the under surface of the diaphragm, at the same time the superior surface of this muscle may contract adhesions with the base of the lung. In such cases the purulent urine and pus from the left kidney is discharged by expectoration.

The contents of the pelvis commonly spread in another direction; the pouch is perforated in its back part, and gives passage to the purulent urine and pus into the extraperitoneal cellular tissue, when they find their way to the crural arch, and very frequently form an urinary abscess in the lumbar region. When these urinary and purulent collections take place, should peritoneal or other inflammation of neighbouring organs, which hastens death, not set in, one or more fistulæ will be established in the lumbar region, or they may open into the colon.

The contents of the pelvis and calices may be arranged as follows:—

1st. Muddy urine, with more or less mucus. Allowed to stand, it throws down a sediment, composed in a great measure of matter, which presents under the microscope a large number of mucous or pus globules, while it has the general aspect of a semi-transparent jelly.

2d. Pus mixed in greater or lesser quantity with urine, and sometimes with blood almost without a trace of urine. It is more or less flocculent or glairy, and becomes very alkaline by putrefaction or the developement of ammonia.

In other cases the pus, from long retention in the pelvis and calices, is changed into a milky or yellowish substance, of the consistence of soft cheese. If, after retaining this substance in water, we examine it with the microscope, purulent globules will be seen in small numbers, with a much greater quantity of granules, which are probably a species of detritus from the pus globules.

3d. Blood, or several of its elements in variable proportions. In some cases the microscope detects the existence of blood globules, where the presence of blood is not obvious to the naked eye.

4th. Calculi, whose forms are moulded by that of the containing cavities: they exhibit branches, and are nearly all terminated by an enlargement. The largest portion of these calculi is nearly always situated in the cavity of the pelvis.

5th. The cavity of the pelvis may also contain sand or gravel, generally loose, and suspended in pus or purulent urine: this sand sometimes forms deposits on the internal surface of the renal pouch, in the form of incrustations, which are generally phosphatic. These saline matters form also with pus, mucus or altered blood, a kind of dark mixture, covering the calculi and the parietes of the pouch.

6th. In other cases, the dilated cavities of the pelvis and calices, contain a whitish solution, which is composed chiefly of amorphous saline matter (phosphate of lime) mixed with animal matter. In one case, Brande found that this whitish solution was composed of nearly pure carbonate of lime. When this white substance is boiled, it immediately blackens, and the residue when incinerated in contact with air, presents a white colour.

7th. Other foreign bodies, acephalocysts, worms, &c., sometimes exist, but much less frequently than calculi in the cavity of the dilated and inflamed pelvis.

In chronic pyelitis the cortical and tubular substances are sometimes inflamed.

In consequence of the accumulation of pus, or of purulent urine in the pelvis and calices, the renal tissues become atrophied, which may be the case with one or both organs, so that the kidney appears to be transformed into a membranous pouch, on which may be seen small portions of the cortical substance. These are the cases which have been considered as throwing out pus from ulceration of the kidneys.

Finally, chronic pyelitis may produce another form of renal atrophy. The kidney of an adult, while preserving its natural form, may be reduced to a size less than that of a new born infant; while the dilated pelvis presents the characters of chronic pyelitis, particularly a considerable thickening of its membranes.

Pyelitis may be produced by a cancerous affection of the kidney, or may co-exist with it. It sometimes accompanies tubercular degeneration. In such cases, after removing by washing, the purulent matter, mixed or not with tubercular detritus, deposited in the pelvis or the calices, there remains a quantity of tubercular matter, adherent to the mucous membrane of these ducts which is frequently ulcerated at several points.

It is rare that an individual dying of pyelitis, chronic or acute, does not present other changes in the urinary passages. In pyelitis there are lesions of the bladder, the urethra, the prostate, and both pelves are almost always affected; but generally there is a remarkable difference in the condition of the two excretory ducts. The alteration is always older or greater in one than the other, without it being possible in a majority of cases to assign the cause of the difference. In the cases of calculous pyelitis, one of the kidneys is greatly changed, while the other, with its excretory duct, is healthy, but hypertrophied.

In reference to lesions of other parts of the body, peritonitis (whether there be perforation of the calices and pelvis or not) is of all consecutive lesions the most common. Inflammations of the large intestine are also very frequent in the latter stages of the disease.

We also see pleurisies, bronchitis, and hepatitis supervene.

Serious alterations of the brain or its membranes are rarely seen, though occasionally cerebral symptoms do occur in the last stages. The developement of these symptoms seems to be due to an alteration in the blood, consequent to the derangement of the urinary secretion.

*Special Characteristics.*—The symptoms of pyelitis, and the accidents more or less varied, which it presents in its progress, either in its acute or chronic state, are so directly and intimately connected with the cause of the inflammation of the pelvis and calices, that practically, it is necessary always to understand the different kinds of inflammation. Now, pyelitis may be arranged into two classes, very distinct. The first comprises those cases of inflammation *induced by foreign bodies*, by one or more calculi, by worms, by acephalocysts, or by retention of urine. In the second class I arrange all those cases of inflammation of the pelvis and the calices, independent of the presence of a foreign body appreciable to the senses; such as the pyelitis following specific inflammations of the urethra and bladder; such also as inflammation produced by the absorption of cantharides, and those, much more serious, which frequently terminate in gangrene, and sometimes supervene on pestilential (*charbonneuses*) diseases, or in cases of purulent absorption in parturient females. Doubtless the diseases in this last group are very different from each other both in their nature and degrees of severity. In placing them in one group, I have desired only to separate them from the diseases produced by foreign bodies.

*Pyelitis from Foreign Bodies.*

Of all the forms of pyelitis, the most interesting, without doubt, is that produced by calculi, (nephritis calculosa, of authors.)

*Pyelitis Calculosa.*

Sand and calculi of different kinds, contained in the pelvis and calices, are the causes of this inflammation.

In opening the bodies of individuals who have died of other dieases than those of the urinary organs, who have presented, in addition to the symptoms of the principal affection, those of acute pyelitis, (pain in the region of the kidneys, one or both, with the excretion of urine surcharged with mucus, without lesion of the bladder or urethra,) I have several times found in the calices and in the pelvis decidedly enlarged, with the walls red and vascular, a fine sand, generally of a reddish yellow colour.

Ordinarily, when the sand is thus deposited in these passages, there exists one or more little calculi, either in the pelvis or in a calix, or in the ureter, whose cavity they contract and obliterate.

At other times, (especially in cases where, during life, symptoms of chronic pyelitis have been observed) instead of urinary sand, we find in the pelvis a white amorphous deposit, similar to the chalky sediment of standing water, and which very often is composed of phosphate of lime. This substance is sometimes so abundant as to fill, with a small quantity of pus, the distended pelvis. In this case the calculus found in the passages is nearly always composed of an ammoniacal-phosphate, and of phosphate of lime.

The small agglomerated crystals, or amorphous matter, more or less dense and insoluble, found in the calices pelvis or ureter, are the frequent cause of pyelitis. Most generally these gravel are irregularly rounded, presenting two facets on which they easily lie, when placed on a moderately inclined plane. Sometimes these gravel appear composed of several nodules. At other times they are stuck together, as if with varnish, or they are irregular and roughened by numerous crystalline asperities, sensible to the finger and visible to the naked eye, or a magnifying glass. When examined with a microscope of moderate strength, these asperities are found to be composed of small crystals. These gravel stones are found nearly always to be composed, when they are yellowish, of uric acid, and when white, greyish or shining, of an ammoniaco-magnesian phosphate. The larger stones are generally found to be composed of ammoniaco-magnesian phosphate, or of oxalate of lime, or of uric calculi encrusted with these salts. The gravel stones of cystine are yellow, and slightly transparent.

In chronic pyelitis the stones, or at least their external layers, are often composed of phosphate of lime, or of the ammoniaco-magnesian phosphate, or of these two salts united, when the urine is alkaline.

The brown gravel, or the greyish brown, are often composed of oxalate of lime, coloured with blood or other animal matter.

The other physical properties of gravel are very variable, even in the same species. Thus, the gravel of uric acid is often formed of so slightly agglomerated crystals, that a little firm pressure will break them ; the centre of these gravel is softer and less compact than their circumference. At other times these same gravel are very solid, and after a blow made with care, a number of concentric layers, composed often of a material more or less pure, tinted more or less deeply, may be observed. When those layers have different compositions, the nucleus is nearly always composed of uric acid, and the outer layers of amorphous crystalline phosphates. We sometimes find these layers alternating, of uric acid and of phosphates. We also observe, but rarely, the nucleus of these gravel composed of a clot of altered blood, or of oxalate of lime.

*Renal calculi*, like the gravel, may occupy the calices, the pelvis, or the neck of the ureter. These calculi present a great variety, in size, form and colour.

The small calculi found in the calices are generally round or oval ; one extremity is often larger than the other. Frequently polished and shiny on their surfaces ; renal calculi may also present inequalities and prolongations like apophyses.

In proportion as the renal calculi increase in volume, and the calices dilate, the tubular substance is flattened against the calculi, and it may be seen from without what part is thinner or thicker in proportion to the projection of the calculus. The extremity of the calculus applied to the cones is flattened, and presents sometimes several facets, which form with each other obtuse angles. These facets are often beveled with a sort of border, and have a neck-like contraction, corresponding to the extremity of the calix. These facets, generally slightly convex, are sometimes irregularly concave; when they are early adapted to the convexity of the conical substance, not yet absorbed.

Once a calculus begins to grow in a calix, it is almost impossible that it should pass into the pelvis, and it will continue to grow. When several cones are included in one calix, the calculus, nearly always increasing in size and obstructing the free course of the urine, compresses the cones, and presents enlargements, which correspond with the depressions in the cones. These tuberosities of the renal calculi are supported by a contraction of the neck; this terminates by a species of queue which projects into the pelvis.

In proportion as a calix distends, it becomes filled with calculous matter or urine; other calices from the same cause are affected in their turn. The compression made on a calix by a calculus in a neighbouring calix, sometimes contributes to the formation of another calculus: the latter being formed in the second calix, the little prolongations or queues touch each other and become united in the pelvis. Generally there are but two calculi thus united: in other cases a greater number become united and form a mass in the pelvis. These calculi have mostly a rounded pedicle, sometimes flat, by which they unite, at right angles, or at angles more or less acute two or three heads which present sometimes a striking analogy to certain bones of animals. We observe also calculi of two calices, placed one opposite the other, uniting in a different manner; the prolongations which are sent into the pelvis unite and leave no appearance of queue in the pelvis. These calculi can never be macerated during life, and it is with difficulty that they can be extracted after death by cutting the indurated and atrophied renal tissue; they are sometimes broken at the necks embraced by the thickened calices.

Finally, it sometimes happens that the pelvis itself is filled with a calculus, uniting itself with others in the calices, forming an irregular mass, differing in shape according to the positions of the calices affected.

Sometimes there is a conical prolongation pointing downwards, on which is attached, at right angles, a branch or appendix as large as the other branch of the calculus. On the extremity of this appendix there are observed facets, more or less numerous, like those described as formed in the neck of the ureter.

At other times, instead of presenting one prolongation, the calculi of the pelvis extend into one or several calices, and assume a very surprising appearance; similar to coral, buck's horns, &c. In all these cases one portion of the calculus, whose dimensions are generally greater than any other individual part, is placed in a vertical position. This, which occupies the pelvis and the commencement of the ureter, frequently exhibits a kind of gutter or canal excavated in its substance. Sometimes this portion of the calculus, placed in the entrance of the ureter, is perforated, as if to permit more readily the passage of the urine from the inflamed pelvis.

This portion of the calculus is sometimes so regularly cylindrical as to have no perceptible depression, at other times it presents a well marked depression, corresponding to the exit of the pelvis. The ureter at this point is nearly always dilated, so much so as to be greater than the pelvis itself.

Sometimes the corresponding extremities of the calculi in the pelvis and in the calices, although contiguous, are not united, but rub against each other: the convex extremity of the one being lodged on the concave surface of the other.

Finally, when renal calculi acquire great dimensions and weigh several ounces, they become less branched than when smaller. The renal substance becomes more and more atrophied without dilatation, the kidney assumes the form of an irregular pouch, which is entirely filled with calculus.

The calculi contained in the ureter are generally small, elongated and irregularly cylindrical. They often obstruct the passage of the urine more completely than larger ones in the pelvis. The exterior of these calculi is often black.

The structure and chemical composition of renal calculi are very various. In general they always present irregular, concentric layers. Sometimes one part of a calculus has a composition just the opposite to that of another part of the same calculus. The internal layer or nucleus is often composed of uric acid; the phosphates form commonly the external layers of moderate sized calculi.

Phosphatic calculi are often friable, and present canals or interstices in their interior.

I have spoken in another part of this work of the *causes* of renal calculi; for the present I would merely say, that, on one side the uric or gouty diathesis, and on the other, the different inflammations of the urinary passages, of the urethra, of the prostate, or bladder, which are easily extended to the kidneys, are the most common causes of *pyelitis calculosa.*

Pyelitis calculosa has been observed at all ages; in the fœtus,* in children,† in adults, and old persons: but more frequently at an advanced age that at any other period of life. It is said that the left kidney is more frequently affected than the right: I am not entirely convinced of this.‡

* Pierre Frank says that a Dutch physician found renal calculi in fœtuses of five and six months (Epitome, trad. Franç, t. v., p. 473, Rétentions hétérogènes); and adds that one of his predecessors, in the Academy of Göttingen, reports the history of two infants, one aged two days and the other eight, who died in convulsions while passing small calculi. Walther (Journ. de Graefe, (t. i. p. 407) found small calculi in the kidneys of a fœtus of eight months.

† Löseke (Obs. Anat. Chirur. Med. nov. et rarior, p. 30) found a renal calculus in a new born infant. Riedlin (Lineæ. Medicæ, 1694, Obs. XXX. p. 312) also found a calculus in an infant about a year old. Dr. Prael, of Brunswick, found several calculi in both kidneys of a female child of six months, (Bulletin de Férrussac, t. x. p. 328.) Harder (cited by Lieulaud, Hist. Med. t. i. c. 271) mentions an infant of three months who suffered symptoms of nephritis: it died at two years of age, when a large quantity of sand was found in the left kidney, and a calculus in the mouth of the ureter. M. Wackenroder (Neue Jahrbuecher der Chemie und Pharmacie, B. VIII., S. 407: B. IX. S. 7 et 67) analysed a calculus found in the kidney of a seven weeks fœtus. We read in the Ephemerides des Curieux de la nature, t. iv., p. 544) that renal calculi were found in an infant of thirty weeks.

‡ Boerhaave (Praelectiones ad instit. p. 252) thinks, that when but one kidney is affected, it is generally the left one. Charles Lepois (Obs. de Morbis a seros. Colluvio, t. iv. c. 2, Obs. c.) says, that of a hundred subjects affected with nephritis calculosa, more than four-twentieths have the left kidney affected; a proportion which appears much exaggerated. Hoffman

The deposit and aggregation of salts in the calices and pelvis are rendered easier not only from certain constitutions of the urine, but also by the accidental presence in this fluid, of foreign animal matters, such as blood, mucus, pus, &c. Thus pyelitis is seen to occur after wounds of the pelvis, blows on the lumbar region, or after powerful contractions of the muscles of the lumbar region, &c.

Calculi (and under this name I include all gravel) may occupy one or more calices without being united, and without presenting any obstacle to the passage of the urine into the ureter: in this case, inflammation is generally seated in parts contiguous to the calculi. While, on the contrary, calculi composed of several branches, occupy both calices and pelves, and obstruct the passage of the urine.

When the renal calculus, situated in the vicinity of the commencement of the ureter, presents a depression, urine and the materials secreted by the inflamed mucous membrane can with greater facility pass through the ureter to the bladder; and the kidney preserves its ordinary dimensions, or at least is not materially enlarged. If the calculus is situated in the entrance of the pelvis or in the ureter, at a greater or lesser distance from its opening, and obstructs it completely, pyelitis is often general, and all that portion of the ureter situated above the obstruction is more or less inflamed and distended with urine mixed with mucus, pus, or blood. There is then a considerable enlargement of the kidney, especially when the disease has existed some months.

The symptoms vary with these several conditions of calculi or gravel.

*Symptoms.*—When gravel or small calculi pass from the calices

(F.) Med. Ration., t. iv., p. 1, sect. 3, cap. 8. S. 7, de febre nephritica) Morgagni (de sed. et causis morb. Epist XL. art. 13,) P. Frank, (Traité de Médècine practique, trad. Franç. de Goudareau, tom. v. p. 474,) Hiesewetter (de lithiasi sinistro quam dextro reni magis infestâ, Halle, 1876,) Earle (Med Trans., t. xi., p. 216, 217 et 27,) Richter (Speciale Therapic, t. iv. p. 483,) Haller (Elementa Physiologiæ, t. vii. p. 368,) Voigtel (Path. anat, 13. iii., S. 188 et 207,) have also thought that the left kidney was more frequently affected than the right. Coiter pretends on the contrary, (extern. et intern. princip., etc. observationesque variæ., Obs. xxiii. S. 3,) that the right kidney is most frequently affected. I repeat that my mind is not made up, the numbers reported not being enough to form a conclusion upon.

into the pelvis, and frow the latter into the ureter, and afterwards into the bladder; the patients suffer ordinarily more or less sharp, and sometimes very acute pains; even before any inflammatory action has been set up. But if a calculus is too large to pass into the ureter, or from the latter into the bladder, it causes derangements, which, in proportion to their acute or chronic characters, present four principal stages.

*First Stage.* (Nephritic colic with suppression of Urine.)—Acute pain, sharp or pungent in the region of the kidney affected, descending to the bladder, and sometimes accompanied with a chill of greater or lesser intensity. Urine scanty, and passed drop by drop, with a sensation of burning, sometimes with small pieces of gravel and a little blood.

The pulse small, depressed, afterwards frequent and full, generally after vomiting once or more, and a feeling of fainting. If the gravel is not expelled the next day or the following days, the greater part of these symptoms continue ; and if the calculus does not entirely obstruct the urine, there is always discharged a quantity of blood and mucus. In cooling, the mucus appears in the urine under the form of soft flakes, like pieces of cotton which are afterwards deposited at the bottom of the vessel. The blood globules, easily distinguished by the microscope, form a light layer on the surface on the sediment.

These first symptoms may cease immediately on the passage of the calculus into the bladder, the urine which had been temporarily charged with blood or mucus, becomes natural, if it be not mixed with pus or mucus in passing through the ureter or bladder.

*Second Stage.*—(Mucous urine.) If, on the contrary, one or more calculi, even small ones, remain in the pelvis or calices, the inflammation, combated or not by art, passes almost inevitably into the chronic state. In this state the pain is not so acute as at first, patients complain only of a sense of weight in the region of the affected kidney. Later, the pain, sometimes none, most commonly dull, may become suddenly very acute, after an effort at motion of the body. It is sometimes worse after a meal ; it is increased by the jolting of riding on horseback or in a carriage ; slight pressure will often induce or increase it. It gradually decreases, and disappears when the patient lies on his side : it sometimes extends in the direction of the ureter, and even to the testicle, and the corresponding thigh and leg, which becomes tumified.

These deep pains, habitual and often independent of febrile action, are sometimes replaced by acute pains, accompanied by other phenomena peculiar to the *exacerbations* of the chronic affections. These pains, sometimes analogous to those of nephritic colic, extend often along the course of the ureter, with retraction of the testicle and swelling of the thigh on the same side. In these exacerbations, blood is discharged with the urine, becoming red and coagulable by heat.

The urine less red than in the first stage, nearly always contains, at least in some discharges, a considerable quantity of mucus, which is deposited on cooling and standing.

Occasionally the urine is bloody, at other times perfectly clear, especially after free imbibition of watery fluids. These different appearances of the urine may sometimes be observed in the same person in the space of twenty-four hours. By cooling, different matters, uric acid or salts, are deposited with the mucus ; blood and mucus are often on the surface of the sediment.

Lying on the abdomen or on the side opposite to the diseased, when one kidney alone is affected, standing, the effort at stool, coughing, sneezing, a deep inspiration, &c., and sometimes the heat of the bed, increase the pain in the kidney. Again, this pain may be very slight, even when there is one or more calculi in the calices or pelvis.

When the gravel which produces these symptoms is composed of uric acid, (and this is most commonly the case,) the urine is acid, and the sediment presents rhomboid crystals of a reddish yellow. Filtered, it becomes slightly muddy when nitric acid is added; this precipitates a quantity of uric acid or albumen, mixed or not with globules of blood.

When the gravel is phosphatic, the urine alkaline and mixed, at the moment of emission, soon becomes clear by the addition of nitric acid, and again cloudy on the addition of an excess of this acid, if it contains albumen, or blood, or pus.

*Third Stage.*—(Purulent secretion, without a renal tumour.)

To the two first stages mentioned, a third succeeds, more serious, when one or more calculi are retained in the pelvis or calices :—irregular chills, which increase towards evening, and are frequently renewed, especially after a meal. Patients suffer in the region of the kidneys with various other morbid sensations, sometimes of cold, which extends down the corresponding member.

The urine, sometimes bloody, more frequently whitish and troubled, deposits, on standing, a purulent sediment, a milky white or slightly green compound, chiefly of pus or of salts precipitated from the urine.

A discharge of blood is sometimes the first striking symptom of the disease, especially when the pelves of both kidneys contain calculi; afterwards the urine becomes troubled and purulent, is discharged frequently and in small quantities at a time; occasionally the patients get better, the urine continuing purulent, with or without a mixture of gravel.

Finally, at periods of longer or shorter duration, patients suffer exacerbations, characterised chiefly by increase of pain in the renal region, by the diminution or suppression of the secretion of urine, a disposition to vomit, vomiting, fever, dryness of the tongue, &c. When death is about to occur, the vomiting persists, the pulse becomes more and more feeble, and the limbs become cold. The cessation of vomiting, diminution of pain and of the prostration, with the re-establishment of the urinary secretion, indicate on the other hand a species of convalescence; but these symptoms will inevitably occur again and terminate in death.

*Fourth Stage.*—(Purulent urine and renal tumour.) The fourth stage is characterized by a tumour (when one kidney is alone affected) in the lumbar region, generally projecting and fluctuating, produced by the accumulation of pus in the pelvis and distended calices. The tumour situated in one of the loins corresponds (when it is in the right side) in its superior part with the inferior extremity of the kidney, under which it is formed. When the tumour has acquired considerable size, its inferior portion may extend to the crest of the ilium, or to the iliac fossa or hypogastrium.

We have seen tumours formed in the pelvis and dilated calices filled with purulent matters, which weighed from ten to sixteen pounds.

In consequence of the developement of these tumours, the lumbar region becomes deformed and enlarged on the diseased side. This enlargement, sensible to the first glance of the eye, in the greater number of cases, may be easily observed by comparing the size of the loin (indicated on each side by a line drawn from the vertebral column to the extreme limit of the trunk) with that of the other side.

By percussion the tumour yields a flat sound, behind, and nearly always in front, if the colon situated before and within the pouch on the right side, and behind on the left side, be not distended by gas. When the tranverse colon separates, at least to a certain extent, the superior extremity from the edge of the liver, (when it is not yet very large,) its limits may be defined by the touch and by percussion; but when the tumour is large, the tranverse colon being doubled and pressed downwards, the tumour having contracted adhesion with the inferior surface of the liver, it then becomes continuous with the liver, which it simulates, or resembles when it is enlarged or contains cysts.

When the renal tumour has acquired a certain size, it appears nearly always nodulated, and composed of several lobes. In this condition fluctuation is perceptible, and it is easy to decide that the tumour is composed of a pouch distended with a fluid.

The pain is never acute in the fourth stage in the intervals of the exacerbations; but it is increased by the pressure of the clothes, or that of the hand applied over the region of the kidney.

The pain is easily produced when, after placing one hand on the anterior surface of the tumour, we press forward with the other applied to the lumbar region. Sometimes pressure from behind diminishes the pain, while that in front increases it. Walking increases the pain, especially when it is rapid, and accompanied with certain movements of the body. Some patients have said that they were conscious of the motion of stones in the distended kidney, and physicians speak of perceiving a peculiar feeling and rubbing in the tumour when pressed upon by the hand.

In the fourth stage the urine is bloody or purulent at every emission, especially when the urine comes from the diseased kidney. The urine is more frequently purulent than bloody; it is only generally in the exacerbations.

At the same time great variations are observed in the physical and chemical properties of the urine emitted. When purulent urine, coming from the inflamed pelvis, being but imperfectly retained in that cavity, it is mixed in variable proportions with the urine of the kidney. This fluid presents in the same day very different appearances, so that in confining ourselves to the examination of one or two emissions, we are liable to great error. I have sometimes seen, in calculous pyelitis, the urine discharged at a particular hour of the day, filled with blood or pus; and at another period in the same patient it would be quite natural; this can only be explained by supposing that the urine comes alternately into the bladder from the diseased and from the healthy kidney. We should always be on our guard in this respect, and not be deceived by a single examination of the urine in the twenty-four hours.

I have already stated that the urine is coagulable by heat and nitric acid, when it contains a certain quantity of pus, but the amount of the albuminous coagulum is not in proportion to the amount of pus mixed with the urine. In certain cases of calculous pyelitis, a considerable quantity of albumen is observed in the urine, while the proportion of globules of pus is very small; while on the other hand urine containing much pus affords but little coagulum, only a light milky tint, after filtering, and applying heat and nitric acid.

When the passage from the pelvis, distended with pus and urine, is entirely closed, the urine passed by the patient may for some days be entirely natural or mixed with pus; while the tumour will continue to enlarge. The renal pain then increases; fever is excited; retching, and even vomiting may occur. Should the urine find a passage through the ureter, the patient will in a short time evacuate a large quantity of urine, which will be accompanied by a sensible diminution of the renal tumour.

If, however, the retention continues, the kidney will be perforated on its posterior surface, either into the peritoneum or intestines. (*See Fistulæ Renales.*)

In other cases still more serious, the emission of urine may be suspended during several days. When complete retention of urine is prolonged, the patient will die, either from a double pyelo-nephritis, from perforations of the kidney, or from cerebral derangement. This retention has been observed in cases of double calculous pyelitis, and in cases where both ureters were obstructed by one or more calculi.

*Fifth Stage.*—(Atrophy of the kidney without purulent secretion.)

Finally, in calculous pyelitis, it may happen that the mucous membrane of the pelvis and calices in contact with a large calculus, becon es so much thickened and indurated, that it will secrete no more or very little pus. In this case the dilated pelvis and calices form with the substance of the atrophied kidney a species of shell, which fits more or less exactly over the calculus. If the opposite kidney is in a good state, this condition, however well marked, may not be suspected during life. Patients will suffer little or no pain, and no pus will be found in the urine. But these patients will die in a few days should calculus obstruct the other ureter, and cause a complete retention of urine.

I may add, that in other cases death will occur without any real retention of urine, probably in consequence of serious derangement of the urinary secretion, or of the suspension of this function. (*Ischuria renales.*) It appears, also, that complete suppression of the urinary secretion may take place when only one of the ureters is obstructed by the calculus.

In several cases, indeed, where the cortical and tubular substances of one kidney were almost destroyed, the urinary secretion has been observed, nearly as abundant as usual, on account of the increased activity or increased size of the other! I will only add one word on certain rare varieties of calculous pyelitis. It is well also to be aware that in consequence of displacement of the kidneys, the seat of pain in calculous pyelitis may be in the umbilical region, or in the hollow of the bony pelvis.

Some authors speak of the symptoms of pyelitis calculosa occurring periodically. Pyelitis calculosa seldom exists alone; it is nearly always associated either with other lesions of the urinary organs, or with lesions of some other apparatus. I will point out shortly the most common and the most important of these *complications*.

*Diagnosis.*—Calculous pyelitis in its various forms may be confounded with a great number of other diseases. Many symptoms of inflammation of the pelvis are, indeed, observed in other affections: thus a mucous or purulent secretion takes place in inflammation of the ureter, bladder and urethra; the pain in the loins exists in lumbago, in nephralgia, &c.: finally, if a pain in the lumbar region be one of the signs of a collection of pus in the dilated pelvis and calices, other lumbar tumours have neither the same origin or the same seat.

1st.—*Lumbar pain* is experienced in different species of acute nephritis, in hydatids of the kidney and of the ureter, in nephritic colic, in retention of urine without inflammation, in lumbago, in certain diseases of the spine, in psoïtis, in aneurisms of the descending aorta, in gestation, in certain diseases of the uterus and its appendages, in some cases of partial peritonitis, and in inflammation of the cellular tissue outside of the peritoneum.

2d.—*In nephritis* the pain is often so much like that in calculous nephritis, that we must resort to other characters to distinguish the affections: in pyelitis calculosa the pain is generally more acute. In doubtful cases we must have reference to the presence or absence of mucous or purulent matters in the urine, and to the existence of a tumour in the lumbar region. If these two symptoms are wanting, we may suspect the existence of pyelitis calculosa, when there is a pain in the lumbar region, with fever, exhibiting exacerbations, with an accession of symptoms similar to those commonly observed in nephritic colic.

3d.—Hydatids of the kidneys, when their cysts communicate with the pelvis, are often accompanied with renal pains, which, as in calculous colics, extend down the ureter to the bladder. There is often a passing retention of urine, with violent expulsive efforts, which are sometimes followed by the voiding of pellicles of hydatids, through the urethra. If there exist at the same time, hydatids in the interior of the pelvis and inflammation of the same, there are occasionally discharges of hydatids, renal pains and purulent urine. The excretion of hydatids and purulent urine, takes place where the hydatids proceed from cysts collected in an inflamed bladder. I have seen acephalocystic cysts, independent of the kidney, present themselves simultaneously from the urinary passages and from the intestinal canal, the hydatids being excreted at once per anum and per urethram.

4th.—Pyelitis calculosa with nephritic colic are distinguished from nephralgia by the fact, that in the first the pain is often much more acute and more intolerable, and that it ceases instantly after the emission of a gravel, or on its passage into the bladder from the ureter.

5th.—In the case of retention of urine, complete or incomplete, it is not uncommon to hear patients complain of a pain in one or both lumbar regions : pains so severe as to induce the belief in the existence of nephritis or of pyelitis calculosa, and yet after death no appearance of inflammation will be seen ; the kidneys, more generally pale than red, appear healthy, with the exception of a slight atrophy of the cones. In this case death occurs suddenly from cerebral derangement.

6th.—In lumbago the pain commonly affects both sides at once, and generally with the same degree of severity ; it is always exasperated by the movements of the body ; it is generally without fever, and sometimes preceded by other muscular or arthritic pains ; in pyelitis the pain is experienced nearly always on one side alone, (cases of double pyelitis calculosa are rare,) or at least with unequal intensity in the right and left sides ; it is augmented by contraction of the lumbar muscles, but this augmentation is not to be compared to the pain occasioned by contractions of the muscles in lumbago. Nevertheless it is always easy to distinguish between the pain of lumbar rheumatism and a renal pain. The opinion expressed by Boerhave on the nature of a pain experienced by him in the region of the kidneys, appears to me to be correct.

7th.—We can scarcely confound pyelitis with *neuralgia of the loins*, in which the pain follows the course of the nerves of the abdominal parieties, or along the last ribs. We must also distinguish the renal pains observed in calculous pyelitis, from dorsal or lumbar pains which pass around the trunk, through the base of the chest or of the abdomen, and which are sometimes accompanied with paralysis of the sphincter ani, of the bladder, or of the inferior extremities.

8th.—In caries of the lumbar vertebra the pain is dull, and generally much less intense than in pyelitis calculosa. This caries is distinguished also by the deformity of the lumbar vertebræ, and often by abscess from congestion, either towards the fold of the groin or towards the buttocks ; finally, by a paraplegia, complete or incomplete ; sometimes without mucus or purulent mucous in the urine. At the same time paraplegia often induces retention of urine, which produces chronic inflammation of the bladder, and sometimes of the ureters and of the pelvis : complications in which we find the characters of caries of the vertebræ and of pyelitis.

9th.—In psoïtis, the patient feels a dull pain in the side affected; this pain becomes afterwards more acute, extending from the lumbar vertebræ to the pubis, and the body is bent forwards and towards the diseased side; the movements of the thigh are very painful; and it is very difficult, not to say impossible, to straighten the back when the body is once bent. After this the glands of the groin become enlarged; then a collection of pus is formed under the peritoneum and substance of the muscle, often with swelling of the limb. When this collection of pus is considerable, it forms sometimes a tumour on one side of the bony pelvis, easily detected by the touch and by percussion. We may confound these purulent deposits with inflamed psoas muscle, with calculous pyelitis, and they may open into the bladder.

10th.—Aneurisms of the aorta have been seen to produce lumbar pains similar to those in the kidneys. Occasionally, the aneurismal tumour has been developed on the side of the abdominal cavity without altering the vertebral column (a case in which it is extremely difficult to detect it): and again it has been found on the lumbar vertebræ, and forming a pulsating tumour in this region.

11th.—Nothing is more common, than to hear, *towards the end of gestation*, females complain of pain *in the kidneys*. But these pains, which are situated much lower than the lumbar region, cannot be confounded with those of pyelitis calculosa. I have already observed that pyelitis calculosa and nephritis occasionally coexist with gestation, and that they are a cause of abortion: the examination of the urine will place us right in this matter.

12th.—Certain diseases of the *ovaries*, *partial peritonitis* in the lumbar region, may simulate disease of the kidney, and especially calculous pyelitis, particularly if they coexist with an acute or chronic cystitis; it is very easy to be deceived under these circumstances.

13th.—The same remark may with greater reason be made in reference to inflammation of the extra-peritoneal cellular tissue, in the vicinity of the kidney. This affection may be confounded with pyelitis, when the urine contains no pus or when there is retention of the matter secreted. On the other hand, when the lining membrane of the bladder is more or less inflamed, the presence of globules of pus in the urine may induce a belief in the existence of inflammation of the pelvis, in the case of peri-nephritis complicated with cystitis. In this last case the diagnosis is never clear.

14th.—Inflammation in a circumscribed point of the ascending or descending colon, may in certain cases simulate calculous pyelitis; but there is nearly always functional derangement of the intestines, especially alternate diarrhœa and constipation, accompanied with the discharge of gas. Otherwise, in calculous pyelitis, the urine is always mixed with a quantity of pus, when the connection between the pelvis and the bladder is not entirely obstructed; in colitis, the fæcal matters are often mixed with blood or mucus. Though we sometimes see these two affections exist at the same time.

15th.—I have occasionally seen hysterical females suffer in the region of the kidney, on one side, pain like that of nephritic colic; but in this case, the urine was pale, very watery, and perfectly transparent.

16th.—Finally, the source of pain may be felt in the wrong kidney. I have seen a patient with calculi in the right kidney suffer the pain in the left.

Cases are cited also where patients have not complained of pain in the kidneys, (pyelitis calculosa latens).

The *secretion* and *excretion of pus* or of purulent mucus in the urinary passages, may exist not only in pyelitis but also in other diseases, either of the urinary apparatus or of other organs. Pus from the extra-peritoneal cellular tissue, the psoas muscle, the ovary, &c., either in the pelvis, in the ureter, or in the bladder; the urine becomes charged with pus from acute or chronic cystitis and simulates disease of the kidney, as also disease of the kidney may simulate one of the bladder.

We have said that when there is neither pain nor swelling in the region of the kidney, the examination of the urine will enable us to distinguish between pyelitis and cystitis; that in inflammation of the pelvis and calices, there is dysuria with a deposit of true pus; that in catarrh of the bladder the urine is glairy and viscous. This remark is true in a certain number of cases; but I have already observed that the glairy appearance may be caused in renal pus by the presence of a certain amount of alkali in the urine, and that the pus of cystitis is not always glairy.

The presence of mucus or pus in the urine, unaccompanied with pain in the bladder, is not characteristic of pyelitis calculosa. To suppose that pus comes from the pelvis or calices, occupied by one or more calculi, there must have existed more or less pain in the upper region of the kidneys.

It is pretended that pus from the kidney is evacuated after the the urine; it is certain that it is evacuated, mixed with the urine, but in greater quantities at the end of the emission, whether it come from the bladder, ureters, or kidney.

I will not now speak of the diagnosis of pyelitis calculosa from critical and metastatic excretions of pus with the urine; I will speak of this in another place.

It is more difficult (on account of the rarity of the case) to demonstrate the true seat of suppuration, when the pus, produced by pyelitis or an extra-renal abscess, passes under Poupart's ligament on the buttock or from the vicinity of the anus. Sometimes the discharges, gravel, generally urates, mixed with the purulent matters which are discharged by the fistula, decide the character of the lesion.

The discharge of purulent fluids, with the odour of urine, or containing urea or uric acid, is pathognomic of renal abscess in the loins.

We have seen pus from an inflamed pelvis discharged with the stools, in consequence of fistulous communication between the dilated pelvis and the duodenum or the ascending colon, or even with the rectum, when the kidney is misplaced.

(To be continued.)

